# Current Management of Conjunctival Melanoma Part 2: Treatment and Future Directions

**DOI:** 10.4274/tjo.galenos.2020.22567

**Published:** 2020-12-29

**Authors:** İrem Koç, Hayyam Kıratlı

**Affiliations:** 1 Hacettepe University Faculty of Medicine, Department of Ophthalmology, Ocular Oncology Unit, Ankara, Turkey

**Keywords:** Conjunctival melanoma, prognosis, management

## Abstract

Conjunctival melanoma is a rare disease which requires tailored management in most cases. The mainstays of treatment can be classified as surgery, topical chemotherapy, radiotherapy, cryotherapy, and other emerging treatment modalities. Herein we review conventional approaches as well as more recently introduced treatment options, together with advances in molecular biology in this particular disease.

## Introduction

Conjunctival melanoma (CM) is a rare malignant tumor arising from atypical melanocytes in the basal layer of the conjunctival epithelium and due to its rarity, the treatment is based on evidence from limited series. There is a growing number of recognized clinical and surgical prognostic factors. The current gold-standard treatment of limited CM can be summarized as surgical excision with or without adjuvant therapy. Adjuvant therapy can be classified further under topical chemotherapy, radiotherapy, and cryotherapy. Incisional biopsy is not recommended to avoid tumor seeding and iatrogenic tumor recurrence.^1^ Tailored management depends on the location and extent of disease. Several studies, however, have revealed that patients treated with excisional biopsy alone without adjuvant therapy had higher risk of local recurrence, distant metastasis, or poorer all-cause and disease-related survival rates.^2,3,4^ Additionally, large, diffuse, or multifocal tumors are more challenging in terms of local control rates even when combined with cryotherapy or radiotherapy.^5^

### Surgery

Primary excision of the CM is the mainstay of treatment when a limbal tumor covers ≤4 clock hours or for any tumor with ≤15 mm basal dimension, using a wide excision with 2- to 4-mm margins.^6^ The main surgical principle is the “no-touch technique” with a dry ocular surface to avoid irritation, as described in the literature.^6^ Frozen section biopsy may also be utilized.^7^ In all cases of CM, care is taken to minimize direct contact between the surgical instruments and tumor and different instruments are used for excision and closure to further avoid surgical implantation. Because limbal CM has a potential to invade the cornea and anterior chamber into the sclera, an additional four-step procedure for limbal CM is described in detail. Step 1 includes localized alcohol corneal epitheliorhexis followed by epitheliectomy to remove any corneal component of the tumor and removal of devitalized cells within a 2-mm margin of the corneal lesion. Step 2 is wide resection including the lesion with 5-mm margins, the underlying Tenon’s fascia, and a 0.2-mm deep partial lamellar sclerokeratoconjunctivectomy avoiding disruption of Bowman’s membrane. Step 3 and step 4 involve cryotherapy on the conjunctival edges followed by alcohol application to the scleral base and closure of the wound with partial or complete peritomy creating transpositional conjunctival flaps, respectively.^6^ Some centers perform sclerectomy only when the tumor is found to be attached to the underlying sclera; for other cases, post-excisional radiotherapy is applied in the form of ruthenium plaque brachytherapy of 100 Gy to a depth of 1 mm to all excised CMs, due to formation of post-sclerectomy scars and an area of possible recurrence or intraocular infiltration with sclerectomy.^8^ With this approach, for forniceal or caruncular tumors, adjuvant proton-beam therapy is employed.^8^ Recently, Cohen and O’Day^9^ clarified their surgical approach to circumscribed CM as adopting a “no-touch” technique and complete resection with 2-mm margins, followed by cryotherapy to conjunctival margins at all times. They also discussed reduction of surgical margins and expanding the use of postoperative strontium applicators for less ocular morbidity, mentioning that the strontium applicator is easily applied and removed without surgery, and strontium radiotherapy has fewer side effects than other radiotherapy methods. The reported recurrence rate with this approach was 10% after a median of 59 months. The authors also limited limbal cryotherapy to adherent disease and lamellar sclerectomy to lesions adherent to the sclera.^9^ For corneally displaced CMs, penetrating keratoplasty could be performed at its own risk if there is a suspicion for a stromal invasion but no further.^10^ Remaining large conjunctival defects after CM excision may require buccal mucosal/conjunctival grafts or amniotic membrane transplantation with fornix-deepening measures such as symblepharon rings.^11^ Amniotic membrane grafts in these cases act as a scaffold for conjunctival epithelial migration and healing, reducing inflammation and fibrosis.^12^ As for more extensive measures for more extensive cases of CM, enucleation for CM is rarely performed since this method leaves potentially diseased conjunctiva behind.^13^ Orbital exenteration, which aims for complete conjunctivectomy, currently is reserved for extensive cases which are unmanageable with other surgical modalities, even though the impact of this procedure on overall survival once there is orbital invasion is considered negligible. For tumors thicker than 1 mm, melanoma-related mortality rate is between 33% and 50% despite orbital exenteration, which is thus reserved as a palliative measure.^14^

### Topical Chemotherapy

The ocular surface is an advantageous location in that it is directly accessible to titratable, repeatable, and high concentrations of topical chemotherapy with minimal systemic exposure to the drugs. Topical chemotherapy in CM is especially beneficial when there is a need to treat the whole ocular surface such as in diffuse or multifocal lesions with ill-defined borders.^15^ In addition, the clinically defined pigmented border of the lesion recognized as the tumor edge may not correlate with the pathological borders which cover the amelanotic edges. However, the use of topical chemotherapy as a primary treatment in CM in contrast to Primary acquired melanosis (PAM) has been limited to a subgroup involving superficial and intraepithelial melanoma, and has been shown to be of limited use when there is nodularity or subepithelial nests; therefore, topical chemotherapy for CM is usually reserved as pre- or post-surgical adjuvant treatment.^5,15^ Topical mitomycin C does not readily cross the basement membrane, thus it is contraindicated as a primary treatment in invasive conjunctival lesions. A literature review of topical antiproliferative therapy for CM is summarized in Table 1.^5,16,17,18,19^

A recurrent CM cell line named CRMM-1 and CRMM-2 has been studied by Westekemper et al.^20^ in terms of sensitivity to chemotherapeutic agents and combinations. Among the tested agents, only mitomycin C and cisplatin were found to have a growth inhibitory effect on tumor cells. The expanded results of the same study group revealed that, after 24-hour exposure of CRMM-1 and CRMM-2 cells to the same agents, the combination of mitomycin C and imatinib had an additive inhibitory effect on tumor growth, whereas combinations of imatinib with fotemustine or cisplatin resulted in antagonism.^20^ All-trans retinoic acid had a synergistic effect with mitomycin or imatinib in CRMM-2 but showed antagonism in CRMM-1. Although 24-hour exposure is impractical in the clinical setting, the authors suggested that a combination of mitomycin with imatinib or all-trans retinoic acid could protect the conjunctiva from mitomycin-related side effects.^20^ These recent results encourage the use of combination therapy or novel potential agents as a part of local treatment in CM.

**Mitomycin C: **Mitomycin C is an alkylating agent isolated from *Streptomyces caespitosus* that exerts an antiproliferative effect during all phases of the cell cycle, making it a powerful tool against both proliferating and non-proliferating cells. It primarily acts by forming a covalent bond with DNA, thereby interfering with DNA synthesis. Secondarily, with topical application under aerobic conditions, it generates free radicals and causes lipid peroxidation. In addition, at the immunohistochemical level, CMs and to a certain extent PAM, express NAD(P)H:quinone oxidoreductase, which promotes bioactivation of mitomycin C.^21^ Table 1 lists the studies in which mitomycin C was used as primary or adjuvant treatment for CM.^5,16,17,18,19^

The reported transient or long-term side effects of topical mitomycin C for ocular surface malignancies include limbal stem cell deficiency, punctal stenosis, ocular irritation, conjunctival hyperemia, tearing, punctate keratopathy, blepharospasm, corneal haze, and ocular pain, with the first two being the most serious complications limiting the use of the drug.^16^ Keratoconjunctivitis and punctate keratopathy are mostly expected to be transient, ceasing over several months and related to longer courses of treatment.^17^ As a countermeasure for acute ocular surface toxicity, cycles are given with 1- to 2-week breaks and with artificial tears or mild topical corticosteroids during, between, or throughout cycles.^16,17^ Care should be taken to avoid direct scleral exposure to avoid further complications. There is no clear dose-response curve to predict side effects; even a single drop of mitomycin C can result in chronic tissue alterations in the conjunctiva by an unknown mechanism. Postoperative use should only be initiated when the wound is properly healed and should be commenced only when surgical margins are proven negative for invasive melanoma.^5,15^

**Interferon-alpha-2B (IFN-α2b):** Interferons are a group of glycoproteins whose antitumor activity is derived from increasing the length of cell cycle, depleting essential metabolites, direct cytotoxicity, modifying expression of cell surface antigens, and induction of antibodies against tumor cells. Data on the ocular use of IFN-α2b for ocular malignancies are mainly derived from studies of ocular surface squamous neoplasia with administration in the form of topical drops or subconjunctival/perilesional injections, and number of studies on its use and effectiveness in CM are limited. When used topically for ocular surface neoplasias, interferons are well tolerated with no or limited ocular surface side effects, such as mild conjunctival hyperemia or follicular keratoconjunctivitis. Perilesional injections might result in systemic side effects such as flu-like symptoms, overnight fevers, and myalgias that respond to acetaminophen. More recently, neoadjuvant intralesional IFN-α2b application has been suggested by Kim and Salvi^22^ for immunoreduction of CM in the hope of better definition of surgical margins and lower local recurrence rates. A review of the literature involving topical IFN-α2b eye drops for CM is summarized in Table 1.^5,16,17,18,19^

**Others: **Peroperative use of sodium hypochlorite or alcohol during excision is practiced in some centers to reduce the risk of dissemination. Sodium hypochlorite in 0.5% concentration with dilutions up to 1/4 and exposure of at least 3 minutes was shown to be cytotoxic to CM cell line (CM2005.1) in vitro, with comparable cytotoxicity to 99% ethanol.^23^ The side effects must be tested in humans.

In terms of adjuvant local intervention, in a recent report studying 2D and 3D cell cultures of CRMM1, CRMM2, and normal conjunctival epithelial cell lines, electrochemotherapy has been suggested as a treatment modality to enhance the antitumor activity of bleomycin, but not mitomycin C and 5-fluorouracil.^24^

### Radiotherapy

The use of radiotherapy for CM can be grouped as internal and external, depending on the mode of application. Radiotherapy currently constitutes a complementary approach as adjuvant treatment to surgical excision of CM. It can be used as a palliative measure solely in the most advanced cases who cannot tolerate exenteration, have surgically unresectable lesions, or tumors irresponsive to other treatments.^25,26^ In postoperative adjuvant settings, it should be used after the wound is completely healed.^27^

**Internal radiotherapy (brachytherapy):** Plaque brachytherapy for epibulbar tumors can be applied with I-125 and Ru-106 isotopes or with Sr-90.^27^ For CM, most recent reports exist on brachytherapy with Sr-90 and I-125.^3,28,29,30,31^ Additionally, Kenawy et al.^8^ have reported their current use of adjuvant Ru-106 plaque for deep invasion with a dose of 100 Gy at 2 mm until 2006 and 100 Gy at 1 mm since 2006, instead of sclerectomy or cryotherapy, resulting in improved local recurrence rates. Plaque brachytherapy with I-125 also poses an adjuvant treatment option in CM when there is corneoscleral involvement. In a study including 5 CM cases with histopathological evidence of scleral and/or corneal stromal involvement that were treated with a 15-mm I-125 plaque for residual disease with 100 Gy at 1.5- to 2.5-mm depth, there were no new local recurrences after a mean 23.4-month follow-up with no intraocular complications. Additional reduced vascularity and inflammation at the brachytherapy site in all patients was noted as a secondary gain.^31^

In a series of 19 bulbar CMs with TNM stage pT1c or less, treatment was carried out as surgical excision avoiding sclerectomy, followed by adjuvant I-125 plaque brachytherapy at a dose of 100 Gy and depth of 1.5-3.0 mm. No local recurrences at the treatment site were observed after a mean 41.3-month follow-up with side effects limited to the perioperative period.^30^

For CM in more challenging anatomical locations such as palpebral conjunctiva or fornix, external beam radiotherapy, proton beam therapy, and even I-125 plaque application have been described.^27^ With this method, a stainless steel shield positioned in the perilimbal position and a dose of 55-60 Gy over 5 days yielded effective local control in 13 of 14 patients over 11-227 months of follow-up (median: 13 months).^27^

Lommatzsch et al.^28^ applied Sr-90/Y-90 brachytherapy in 10-Gy fractions until the applied total dose was 150-200 Gy, depending on the thickness of the lesion. The local recurrence rate was 19/81 in this cohort of CMs, where 46 had adjuvant or primary plaque brachytherapy and 3 had adjuvant external beam radiotherapy. Their series reported a total of 23.5% local recurrence rate after a mean of 66 months regardless of the mode of treatment.^28^ In their nationwide study of 194 CMs, Missotten et al.^3^ reported local recurrence rates of 67% with excision only and 26% when Sr-90/Y-90 brachytherapy was performed in combination with surgery, with median follow-up of 6.8 years. Twenty patients with bulbar CM undergoing Sr-90 beta irradiation with a handheld applicator with 5 fractionated doses of 50 Gy to the scleral surface as an adjuvant treatment also had successful results in terms of a local control rate of 90% after a median of 59 months with mild local complications and no cataracts.^29^ The authors define the indication for this treatment as positive deep surgical margins.^29^

**External radiotherapy:** The use of external beam radiotherapy (EBRT) in CM has been reported in patients who cannot tolerate surgery due to old age and bad health, as an adjuvant therapy, and with lesions too large for resection.^26,27^ Some studies justify the use of postoperative EBRT with a median of 60 Gy when there is aggressive histology, microscopic perineural invasion, advanced-stage disease, or positive margins in malignant lesions of the conjunctiva and eyelid.

Proton beam irradiation is another method of external irradiation which is more selective to the target tissue with less collateral damage than EBRT. Currently, some centers have expanded the use of proton beam radiotherapy in CM to include patients with tumors >1.5 mm in thickness, diffuse or multifocal disease, presence of PAM, forniceal or caruncular lesions, and positive histopathological margins, applied as 36 Gy in 6 fractions 2 weeks after excisional surgery. With this method, 5-year recurrence free survival was reported as 81%.^32^

Wuestemeyer et al.^25^ studied proton beam therapy in 20 patients as an alternative to orbital exenteration. Most tumors were stage T3, and all had forniceal or caruncular location except 2 bulbar tumors. After excisional biopsy and conjunctival mapping, 31 Gy in 6 fractions and an additional 2 fractions up to 45 Gy were applied. The median follow-up was 34 months. The recurrence rate was reported as 30%. As a result, proton beam radiotherapy was proposed as an alternative to exenteration for T3 or diffuse T1 and T2 tumors. The most frequent notable complications were dry eye (95%), focal cataract (35%), and limbal stem cell deficiency (20%).^25^

In another study where proton beam radiotherapy was used more liberally in a larger cohort of 89 patients with CM from stage T1c/d to T3, the 5-year cumulative rate of eye preservation was 69% and the estimated overall 5-year survival was 71%, thus offering proton beam radiotherapy as an alternative to orbital exenteration in T2 and T3 tumors.^33^ Thirty-six (41%) patients were previously treated, and 29 patients (33%) developed local recurrence.^33^ The most common side effects were sicca syndrome in 27, secondary glaucoma in 10, and limbal stem cell deficiency in 7 patients.^33^

### Cryotherapy

At present, adjuvant cryotherapy is described as one of the stages in excision of CM, as previously mentioned. The freezing process in cryotherapy ultrastructurally mimics the damage of a thermal burn, which causes shedding of the superficial epithelium from the substantia propria with the superficial atypical melanocytes, in addition to direct damage to tumor cells due to ice crystals, which cause cell lysis. It is advised to target the very superficial melanocytes or the small number of melanocytes potentially left behind in the deeper layers of conjunctiva after excision, and not to treat the nodular portion with cryotherapy only.^34^ The use of cryotherapy aids in reduced exenteration rates in unifocal CM, but multinodular CM has metastatic rates as high as 45% when surgery is combined with cryotherapy. It is also advisable not to perform cryotherapy on bare sclera but to prefer alcohol application to avoid potential scleral melt. To overcome inadvertent tissue damage and enhance the effectiveness of cryotherapy, Finger introduced “finger-tip” cryotherapy probes which formed more homogenous burns over larger areas and covered flat target areas more effectively with less chance of missing the tumor.^35^

Application of cryotherapy has been shown to effectively reduce local recurrence rates in a series by De Potter et al.^36^ In their cohort of 68 histologically proven CMs, treatment modality was the only factor associated with local tumor recurrence, which was reported at a rate of 68% with surgical excision only and was reduced to 18% when surgery was combined with cryotherapy over a mean 7.5-year follow-up. Thus, it still remains one of the most effective adjuvant modalities in current practice.

### Other

The molecular biology of CM and biological similarities to cutaneous melanoma has implications in its treatment. Vemurafenib is a V600E mutation-specific BRAF inhibitor that has been suggested as a treatment of metastatic disease.^37^ *In vitro* studies of vemurafenib, dabrafenib, a MEK inhibitor (MEK162), and an AKT inhibitor (MK2206) showed that the combination of the latter two drugs had a synergistic effect in the inhibition of cell proliferation, but a *BRAF* wild-type and *NRAS* mutated cell line was irresponsive to *BRAF* inhibition.^38^

For cutaneous melanoma, *BRAF* mutation has been a point of interest for potential targeted therapy in metastatic melanoma; however, there are only a few publications consisting of single reports regarding BRAF with or without MEK inhibition in CM. Among these, one reported 12-month recurrence-free, stable, initially metastatic CM with dabrafenib (BRAF inhibitor) combined with trametinib (MEK 1 and 2 inhibitor) in a 70-year-old male^39^, and 2 reports described complete regression of metastatic CM and non-metastatic CM with trametinib combined with vemurafenib or dabrafenib, respectively.^40,41^ Kiyohara et al.^42^ reported 2 cases of metastatic CM, one of which was initially managed with vemurafenib for metastasis, which was later switched to dabrafenib with trametinib due to keratoacanthoma-like eruptions thought to have been caused by vemurafenib, but the patient was lost after 24 months of follow up. The other patient had been followed successfully for 6 months with dabrafenib with trametinib without local recurrence. These data and the non-uniform results provide little on which to make generalized assumptions, but it is clear that BRAF inhibition in BRAF-mutated cases, particularly with MEK inhibitors, is one of the most promising targeted therapies for CM.

Immune checkpoint inhibitors are novel drugs for targeted therapy, also used in cutaneous or unresectable cutaneous melanoma, which act on receptors of activated T lymphocytes and facilitate recognition of tumor cells by the host immune system. A recent report of 5 patients with metastatic CM examined the results of immunotherapy with programmed cell death 1 (PD-1) inhibitors. Four patients had received nivolumab and one received pembrolizumab as PD-1 inhibitor. The patients treated with nivolumab were disease-free after 36 months. The patient treated with pembrolizumab showed progression after 11 months and was switched to another therapy.^43^ Considering a recent analysis by Cao et al.^44^ in which PD-ligand-1 was detected in 19% of primary CMs, immunotherapy is a potential treatment option for systemic disease. The study also suggested that this expression was correlated with distant metastases and a worse melanoma-related survival.^44^ To predict the success of PD-1 inhibitors, the additional determination of HLA Class I antigen status is recommended, as its expression is found to be independent from PD-1/PD-L1 expression in CM.^45^

In a recent case series of 5 patients, 3 patients with locally advanced CM who refused orbital exenteration and 2 with metastatic disease received multiple cycles of an anti-PD1 agent together with ipilimumab or nivolumab.^46^ All cases showed improvement in local and metastatic CM and complete response was seen in 2 patients, 1 of whom initially had systemic disease.^46^

Another newly proposed potential target is an epigenetic modifier, enhancer of zeste homolog 2 (EZH2), which is highly expressed in primary CM and lymph node metastases (50% and 88%, respectively) but absent in normal conjunctival tissue.^47^ Pharmacological inhibition of EZH2 with GSK503 and genetic knock-down resulted in diminished cell growth *in vitro* and zebrafish xenografts.^47^

Tumor-associated lymphangiogenesis is another potential target for treatment in CM. A study of intratumoral lymphatic vessel density by staining lymphatic vascular endothelial hyaluronan receptor-1 and podoplanin as lymphatic endothelial markers showed that higher intratumoral lymphatic vessel density was correlated with higher tumor thickness and larger tumor diameter, as well as lower recurrence-free and higher melanoma-related death rates.^48^ The same markers were used to compare intra- and peritumoral lymphatic vessel density in C-MIN with and without atypia and in CM. CM showed the highest intra- and peritumoral lymphatic vessel density while none of the C-MIN lesions without atypia showed positive staining for these markers intra- and peritumorally, which implies lymphangiogenesis as an early step in malignancy development, even before invasive stages.^48^ Additionally, non-limbal tumors with tarsus or fornix involvement are shown to have a tendency for higher lymphatic vessel density than limbal tumors, which implies that non-limbal tumors would benefit more from a potential anti-lymphangiogenic treatment.^49^ In terms of comparison of the lymph- and hemangiogenic profile of CM and uveal melanoma cell lines, vascular endothelial growth factor (VEGF)-A, -C, and -D mRNA, and VEGF-A and -D protein expressions were all seen in CM and uveal melanoma cell lines, and they did not differ in lymph- and hemangiogenic potential. This suggests the existence of *in vivo* mechanisms that act on the tumor microenvironment and lead to a preference for lymphatic spread of CM and hematogenous spread of uveal melanoma.^50^

One final putative target for inhibition is the mTOR (mammalian target of rapamycin) pathway, since phosphorylated m-TOR effectors are highly expressed in CM, unlike uveal melanoma where PTEN was responsible for mTOR pathway downregulation.^51^ mTOR pathway inhibition as a potential therapy has been a part of an *in vitro* study where 3 cell lines (CRMM1, CRMM2, T1527A), have been subjected to a BRAF inhibitor (vemurafenib), two MEK inhibitors (trametinib, selumetinib), a PI3K inhibitor (pictilisib), and a dual PI3K/mTOR pathway (dactolisib).^52^ The cell lines differed in their mutational profile which included *BRAF* V600E mutation for CRMM1, *NRAS* Q61L mutation for CRMM2 and *BRAF *G466E mutation for T1527A. As a result, CRMM1 was found to be sensitive to inhibitors of both MAPK (trametinib and only marginally to vemurafenib), CRMM2 was found to be moderately sensitive to pictilisib, and T1527A was resistant to all tested agents; vemurafenib sensitivity was only displayed by CRMM1.^52^ Thus, 2 of 3 cell lines, CRMM1 and CRMM2, which harbored the most commonly encountered mutations, showed significant growth inhibition with pictilisib (PI3K inhibitor). Interestingly, however, this effect was reduced when pictilisib was combined with the downstream mTOR inhibitor, dactolisib.^52^

### Molecular Biology

The most commonly studied and reported mutations found in CM include *BRAF, NRAS*, and *KIT* mutations. Furthermore, the similarities in genetic alterations have suggested a biological kinship between CM and cutaneous melanoma in recent years, which raised interest for the development of potential new therapies.^53,54^

The *BRAF* (v-Raf murine sarcoma viral oncogene homolog B) gene encodes a serine/threonine kinase involved in signal transduction in the mitogen-activated protein kinase (MAPK) pathway. Activating *BRAF* mutations can be found in up to 50% of CM, and among *BRAF* mutation-bearing samples, the ratio of *BRAF* V600E to *BRAF* V600K is nearly 4:1.^37,53,54^ It is debatable whether *BRAF* mutations are of prognostic significance, but a population-based study in Denmark has correlated *BRAF* mutation status with male gender, younger age, sun-exposed tumors (which included bulbar conjunctiva or caruncle), mixed or non-pigmented color, absence of PAM, and CM of nevi origin.^2^

*NRAS* stands for neuroblastoma v-Ras oncogene homolog, and this gene encodes a GTPase promoting proliferative cycle of the cell. Activating *NRAS* mutations can be found at up to 18% frequency and are mutually exclusive with *BRAF* mutations.^38^ Remarkably, *GNAQ* and *GNA11* mutations are virtually nonexistent in CM, which differs from uveal melanoma.^54^ The *KIT* gene encodes a receptor tyrosine kinase which promotes cell survival and growth and is found to be mutated in nearly 2% of CM.^55^ *KIT*-mutated melanomas are shown to be sensitive to imatinib, a tyrosine kinase inhibitor including c-kit. CD117 expression and c-kit immunostaining do not correlate with *KIT* mutation status or copy number; therefore, an analysis of mutational status is advised be performed before commencing to imatinib treatment.^56,57^

A more recent large cohort of 63 CMs demonstrated *NF1* mutations as the most frequent mutation in CM (33%), followed by activating mutations of *BRA*F and *RAS* genes, all of which induce activation of the MAPK pathway.^58^ The authors proposed a genetic classification of CM similar to cutaneous melanoma, including *BRAF*-mutated, *RAS*-mutated, *NF1*-mutated and triple wild-type CMs, implying mutual exclusion of each entity.^58^

As for other mutations that were detected in CM, whole exome sequencing in excised material of 5 CM patients showed that in addition to *BRAF, NRAS*, and *NF1* mutations, CM harbors previously unreported mutations in *EGFR, APC, TERT*, and other cancer-associated genes and the C→T mutation signature consistent with UV-induced DNA damage. The most common chromosomal alteration was 6p gain.^59^ Recent studies of molecular and genetic/epigenetic alterations seen in CM are summarized in Table 2.^37,44,54,60,61,62,63,64,65^

As a contribution to clinical interpretation of the copy number alterations in CM, single nucleotide polymorphism array has been conducted in a multi-center study in 59 CM to study the correlation between copy number alterations and clinical outcome.^66^ Four tumor suppressor genes (*NEURL1, SUFU, PDCD4, C10orf90*) which were affected by deletions of chromosome 10q24.32-26.2 were found to be significantly related to CM metastasis. Deletions of 10q24.32-26.2 were also strongly associated with lymphatic invasion and increasing tumor thickness.^66^

### Conclusion and Future Directions

Even though CM is a rare disease, the potential mortality makes accurate diagnosis and appropriate treatment imperative. The literature data consists mostly of a limited number of studies due to the rarity of the disease. Currently there is an almost uniform approach for initial treatment of limited, focal disease, consisting of excisional surgery and cryotherapy, although approaches to more advanced disease or adjuvant treatment differ between centers. Even with adjuvant treatment, mortality rates can only be reduced to a certain extent. Further classification of CM is still needed for individual prognostic and survival prediction. Genetic and molecular alterations common to CM and cutaneous melanoma make it amenable to studies on targeted molecular therapy. Multi-center and prospective trials would improve our understanding of the biological behavior of this potentially deadly tumor by providing more information about the molecular alterations implicated in the development of the disease and the corresponding targeted therapy.

## Figures and Tables

**Table 1:**
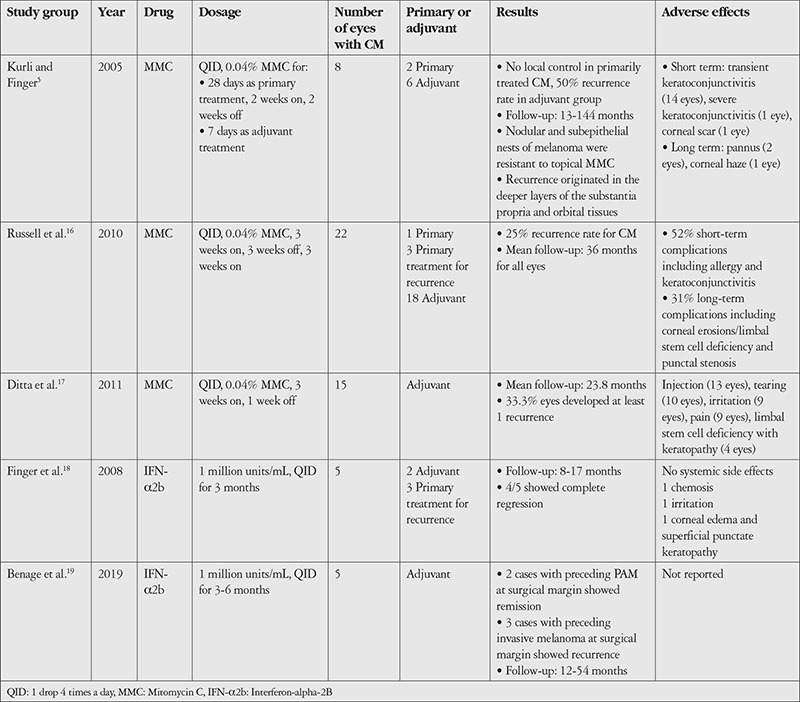
Literature review on topical chemotherapy for conjunctival melanoma (CM). Case reports and studies with less than 5 CMs are excluded

**Table 2:**
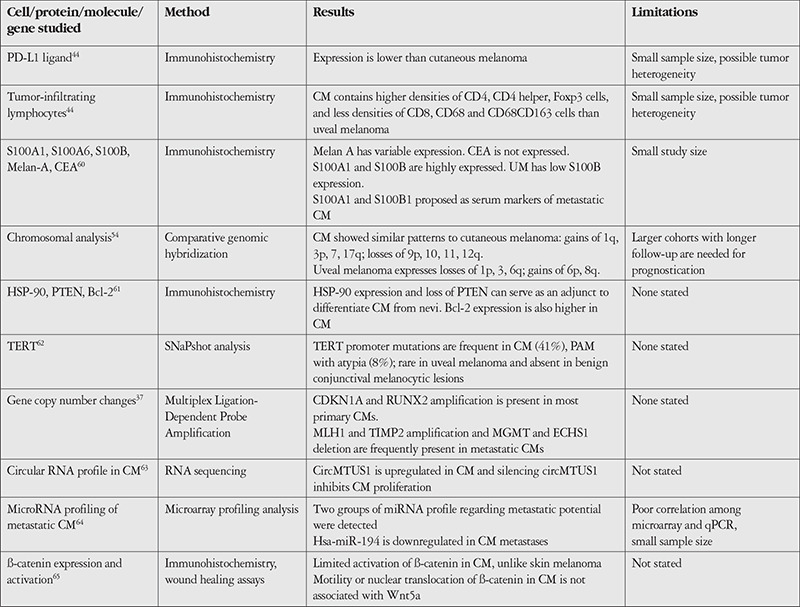
Studies on molecular pathology, genetics, and epigenetics of conjunctival melanoma (CM)
